# Real-world use of difelikefalin in hemodialysis patients at a large dialysis organization in the United States: a retrospective database study

**DOI:** 10.1186/s12882-025-04074-7

**Published:** 2025-03-27

**Authors:** Linda H. Ficociello, Rachel Lasky, Hans-Juergen Arens, Despina Ruessmann, Michael S. Anger

**Affiliations:** 1 Renal Research Institute, Waltham, MA USA; 2 Fresenius Medical Care D GmbH, Bad Homburg, Germany; 3 CSL Vifor, Global Medical Affairs, Glattbrugg, Zurich, Switzerland; 4 Fresenius Medical Care, Global Medical Office, Waltham, MA USA

**Keywords:** Chronic kidney disease–associated pruritus, Difelikefalin, Hemodialysis, Symptoms

## Abstract

**Background:**

Chronic kidney disease–associated pruritus (CKD-aP) can negatively impact quality of life and survival among patients receiving maintenance hemodialysis. Difelikefalin, a selective κ-opioid receptor agonist, is the first medication approved for treatment of moderate-to-severe CKD-aP among patients on chronic hemodialysis. This retrospective database study assessed the real-world safety and effectiveness of difelikefalin across a large US dialysis organization.

**Methods:**

We analyzed de-identified data from 715 adult hemodialysis patients treated with difelikefalin who had a Worst Itching Intensity Numerical Rating Scale (WI-NRS) score (0 = no itching to 10 = worst itch imaginable) assessed before therapy. Patients were classified as having received at least 30 difelikefalin doses over 12 weeks (complete regimen group; CRG) or fewer doses over that time period (incomplete regimen group; IRG). Mean baseline and follow-up WI-NRS scores were compared and potential adverse events evaluated.

**Results:**

Mean (SD) baseline WI-NRS scores were 8.5 (1.7), indicative of severe pruritic symptomatology. In the 22% of patients with follow-up data, mean WI-NRS scores improved by 2.9 points (8.4 [severe] to 5.4 [moderate]; *P* < 0.0001). This mean improvement was more pronounced in CRG patients (*n* = 84; 3.6) compared with IRG patients (*n* = 84; 2.2). Overall, 46% of patients experienced a 3-point reduction in itch severity. Difelikefalin initiation was not associated with changes in rates of nausea, diarrhea, vomiting, headache, or trouble walking. Dizziness and hyperkalemia were infrequent, but statistically significant with increases in dizziness (0.09% vs. 0.20%) and hyperkalemia (2.0% vs. 2.6%) were observed during treatment with difelikefalin.

**Conclusions:**

In this analysis of real-world difelikefalin use in a US hemodialysis population, patients experienced significant reductions in CKD-aP, based on a validated measure of pruritus. Patients remaining on therapy for 12 weeks demonstrated greater symptom reductions than those patients receiving partial treatment. In combination with controlled trials, these data suggest that difelikefalin is an effective and well-tolerated treatment for the management of CKD-aP in adult patients receiving hemodialysis.

**Supplementary Information:**

The online version contains supplementary material available at 10.1186/s12882-025-04074-7.

## Introduction

Chronic kidney disease–associated pruritus (CKD-aP), previously referred to as uremic pruritus, is itching due to kidney disease that can vary in clinical presentation from mild to severe and can affect patient quality of life and survival [1–3]. CKD-aP can be recurrent and sometimes persistent in patients with end-stage kidney disease (ESKD) who are on dialysis.

The pathophysiology of CKD-aP has not been well established and is poorly understood [1, 2]. Historically, it was believed that the deposition of “uremic toxins” in the skin and subcutaneous tissue acted as pruritogens. In recent years, additional pathogenetic mechanisms have come to light. Immune dysregulation can lead to micro-inflammation in the skin, with a resultant increase in pro-inflammatory cytokines and other mediators. Peripheral neuropathy can cause pruritus when diseased afferent sensory neurons are disproportionately activated. Lastly, opioid imbalance with overstimulation of µ-opioid receptors and antagonism of peripheral κ-opioid receptors has also been proposed as a potential mechanism for pruritus.

The prevalence of CKD-aP in hemodialysis patients varies, depending on the population studied and diagnostic tool utilized. In a large international survey of hemodialysis patients in the Dialysis Outcomes and Practice Patterns Study (DOPPS), the prevalence of moderate-to-severe CKD-aP was 37%, with variability noted across countries [3]. In the United States, 33% of patients self-reported moderate-to-severe pruritus [3]. The prevalence of CKD-aP may be under-reported for many reasons, including patients’ unawareness of the link between pruritus and kidney disease, as well as care providers not asking questions about patient symptoms [4].

Several tools exist to measure patient-reported severity of pruritus, such as the Worst Itching Intensity Numerical Rating Scale (WI-NRS) [5]. The WI-NRS asks patients to “describe the worst itch intensity they experienced over the last 24 hours” on a scale of 0 (no itch) to 10 (worst itch imaginable) and has been validated in CKD-aP [6]. In a qualitative study of patients with CKD-aP, a reduction of itch severity of ≥ 3 points was determined to be meaningful to patients [6]. A score of 4–6 is considered moderate pruritus, and ≥ 7 is considered severe pruritus [6].

Historically, treatment options for CKD-aP have been limited to the use of therapies that are not approved by the US Food and Drug Administration (FDA) for the management of this condition. These pharmacologic and non-pharmacologic interventions lack strong evidence supporting their safety and efficacy. Even with the existence of clinical guidelines and specific literature on the management of CKD-aP [7–9], nephrologists have had to rely on approaches based on personal experience. Options used by clinicians have included topical emollients for prevention of xerosis, optimization of hemodialysis treatment, oral antihistamines, neuropathic agents such as gabapentinoids, antidepressants, and non-pharmacologic interventions such as ultraviolet B therapy and acupuncture [10]. The absence of regulatory approval for these treatment approaches and the limited evidence regarding their safety, efficacy, and tolerability from dedicated large-scale clinical trials have led to a large unmet need for CKD, and particularly ESKD, populations [11].

Of the therapeutic options evaluated for the management of CKD-aP, difelikefalin (DFK; KORSUVA, Cara Therapeutics, Inc., Stamford, CT, USA) is the only approved on-label drug to treat CKD-aP. It is a selective κ-opioid receptor agonist that works predominantly by activating the κ-opioid receptors on peripheral sensory neurons and certain immune cells. It minimally crosses the blood–brain barrier and has primary action outside the central nervous system, thereby providing a better safety profile than other opioid agonists [11]. A study on rapid discontinuation of DFK compared to continuation of DFK demonstrated no differences in withdrawal symptoms between groups [12]. It is the first medication approved by the FDA for the treatment of moderate-to-severe CKD-aP among patients with CKD on chronic hemodialysis.

The clinical profile of DFK was established in two phase 3, randomized, double-blind, placebo-controlled trials in cohorts of exclusively hemodialysis patients: KALM-1 [13] in the United States and KALM-2 [14] in the United States, Europe, and Asia-Pacific. DFK was generally well tolerated, and patients showed no sign of dependency in a 2-week discontinuation phase [13]. Pooled efficacy and safety analyses of both trials were also conducted [15, 16]. DFK was shown to lower pruritus severity by at least 3 points at week 12 significantly more frequently than placebo (51% vs. 35%; *P* < 0.001).

The aim of the current database study is to assess the real-world usage, including safety and effectiveness, of DFK in patients with CKD on chronic hemodialysis.

## Methods

### Cohort and data extraction

This retrospective database analysis examined all Fresenius Kidney Care (FKC) patients ages 18–89 years in the United States undergoing hemodialysis, who (1) received at least one dose of DFK before November 15, 2022, and (2) had at least one WI-NRS score before receiving a DFK dose. We intentionally included patients with at least one WI-NRS in our real-world study in order to encompass a broader patient population.

All data were extracted from the FKC clinical data warehouse and de-identified. The study was determined to be exempt under 45 CFR § 46.104(d)(4) by an independent institutional review board (New England Institutional Review Board [NEIRB], Needham, MA, USA; WCG IRB Work Order #1-1650918-1).

### Treatment with DFK

Patients were treated with DFK according to a prescription from their physician, as part of the patient’s routine dialysis care. The FKC clinics utilized a clinical algorithm for the management of moderate-to-severe CKD-aP, with DFK administered at 0.5 mcg/kg intravenously. As this study was retrospective in nature, it had no impact on DFK prescriptions or other therapies.

Two groups of patients were formed based on whether they had completed 30 or more DFK administrations within 74–84 days of the first DFK dose (complete regimen group; CRG) or did not (incomplete regimen group; IRG).

### Assessment of itch

The baseline WI-NRS was defined as the score closest to DFK start (which must have been within 90 days). The 12-week WI-NRS was the assessment within ± 10 days of 84 days after the first dose of DFK. Patients having a 12-week WI-NRS were included in the change of itch severity analysis. On the WI-NRS, moderate itch is defined as a score between 4 and 6, inclusive, and severe itch is defined as a score between 7 and 10, inclusive.

### Adverse events and reasons for discontinuation

Potential adverse events before and after the first DFK dose were obtained from evaluation and nursing notes. Notes were flagged for prespecified symptoms, such as diarrhea, nausea, vomiting, dizziness, trouble walking, and headache, and were manually reviewed to determine whether symptoms were present or absent. Hyperkalemia was defined as serum potassium levels > 5.5 mEq/L. The medical record has a field for medication discontinuation reason that served as the primary source of reasons for discontinuation. In addition, treatment evaluation notes, clinician notes, and nursing notes were reviewed for medication discontinuation reasons when no reason for discontinuation was explicitly noted. Hospitalizations and missed hemodialysis sessions due to any reason were counted.

### Statistical methods

Mean baseline and follow-up WI-NRS scores were compared within patient groups using paired t-tests. T-tests were also used to compare the mean WI-NRS scores of the CRG and IRG at baseline and at follow-up, and to assess the magnitude of change in severity of pruritus between groups. A confirmatory analysis with a linear mixed-effects model was also conducted, which demonstrated similar results as the t-test. Given the consistency between the t-test and linear mixed-effects model findings, the output of the simpler t-test was applied in the results reporting. The percentage of patients achieving 1-, 3-, and 4-point decreases in itch severity was also examined. The number of various adverse events was first considered as a percentage of the total dialysis treatments before and after the first DFK administration within each group (all patients, CRG, IRG, and discontinued patients). The frequency of occurrences before and after the first DFK dose was then compared using χ-square testing. Subgroup analyses of baseline and follow-up WI-NRS scores were conducted based on the use or non-use of concomitant medications (gabapentinoids and antihistamines) and by baseline symptom severity (moderate or severe). Statistical analyses were performed using SAS Enterprise Guide version 7.1 (SAS Institute Inc., Cary, NC, USA).

## Results

### Cohorts

Baseline characteristics of the 715 patients included in the cohort are described in Table 1. On average, patients experienced severe pruritic symptomatology at baseline (mean ± SD WI-NRS: 8.5 ± 1.7) and were an average of 64.7 years old, with a mean dialysis vintage of 4.8 years. The 12-week WI-NRS score was available in the database for 22% of the overall patient cohort. Approximately 40% of patients were Black or African American, 10% of patients were of Hispanic ethnicity, and most had a primary payer of Medicare (45%) or managed Medicare (54%).

**Table 1:** Demographics by group

Characteristic	All Patients (*N* = 715)	CRG (*N* = 295)	IRG (*N* = 420)
Baseline (pre-DFK) WI-NRS score, mean (SD)	8.5 (1.7)	8.5 (1.8)	8.5 (1.6)
12-week WI-NRS score available, *n* (%)	156 (21.8)	84 (28.5)	72 (17.1)
DFK dose, mL, mean (SD)^a^	0.81 (0.24)	0.83 (0.25)	0.79 (0.24)
Female, *n* (%)	345 (48.3)	124 (42.0)	221 (52.6)
Black, *n* (%)	285 (39.9)	113 (38.3)	172 (41.0)
Hispanic, *n* (%)	68 (9.5)	29 (9.8)	39 (9.3)
Age at first DFK administration, years, mean (SD)	64.7 (13.0)	66.1 (12.9)	63.8 (13.1)
Vintage at first DFK administration, years, mean (SD)	4.8 (4.5)	4.4 (4.2)	5.2 (4.6)
Primary insurance, *n* (%)			
Medicare	319 (44.6)	129 (43.7)	190 (45.2)
Commercial	5 (0.7)	2 (0.7)	3 (0.7)
Managed Medicare	388 (54.3)	162 (54.9)	226 (53.8)
Exchange	2 (0.3)	1 (0.3)	1 (0.2)
Self	1 (0.1)	1 (0.3)	0 (0.0)

^a^Supplied as 50 µg/mL. CRG, complete regimen group; IRG, incomplete regimen group; DFK, difelikefalin; WI-NRS, Worst Itching Intensity Numerical Rating Scale; SD, standard deviation

### Patient disposition

**Fig. 1:**
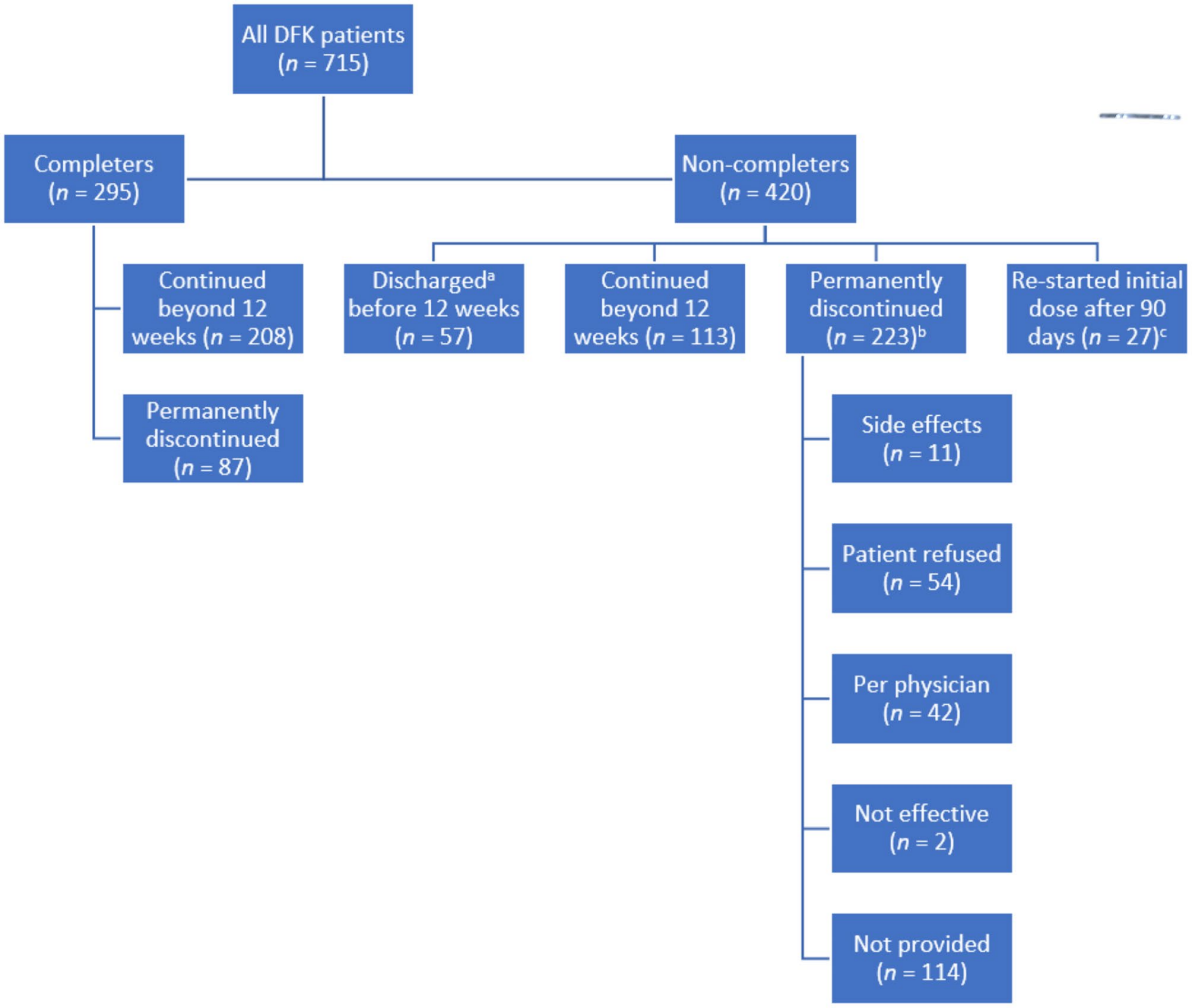
Patient disposition ^a^Reasons for discharge before 12 weeks include death, transplantation, withdrawal from dialysis, transfer out of FKC, and other ^b^Of 223 patients who permanently discontinued DFK, 103 had reasons for discontinuation listed in the database within 7 days of their final DFK administration. Of 120 patients with no discontinuation, notes from the electronic medical record were reviewed, as follows: *Clinician notes*: 1 patient had a note related to DFK during the follow-up period; 0 patients were re-categorized for mention of relevant discontinuation reasons. *Nursing notes*: 9 patients had notes related to DFK during the follow-up period; 2 patients had clearly stated reasons for discontinuation; 3 patients were re-categorized for mention of relevant discontinuation reasons. *Pre-/post-evaluations*: 11 patients had notes related to DFK during the follow-up period; 1 patient was re-categorized for mention of relevant discontinuation reasons ^c^Patients who discontinued DFK who were found to have re-started an initial dose of DFK after the 90-day follow-up period were re-categorized DFK, difelikefalin; FKC, Fresenius Kidney Care

Figure 1 provides information on patient disposition, including 295 patients classified as being in the CRG (completers) and 420 as being in the IRG (non-completers). Most patients in the CRG (71%) continued DFK beyond 12 weeks. In the IRG, 14% of patients were discharged (e.g., death, transplantation, withdrawal from dialysis, transfer out of FKC) before the end of 12 weeks, 33% continued or re-started after the 12 weeks, and 53% discontinued DFK use. Among patients who discontinued DFK, the mean time to discontinuation was 39.6 days, and an average of 13 doses were administered prior to discontinuation. A reason for discontinuation was identified for 49% of patients; reasons included (in order of frequency) patient refusal, physician decision, side effects, and absence of effectiveness. Information regarding the specific factors for patient refusal was not available in the database.

The baseline WI-NRS assessments occurred prior to the start of DFK, with a mean time period of 10 days prior to the first dose. The follow-up 12-week WI-NRS assessments were conducted 84 days ± 10 days after DFK initiation, with a mean time period of 82 days after the first dose. Among patients who discontinued DFK, 44 had baseline and follow-up WI-NRS assessments. Mean WI-NRS scores improved in all discontinuation groups except those in which “side effects” were the reason for discontinuation (mean at baseline, 8.0; mean at follow-up, 10). The “not provided” group was the largest (*n* = 31), and their itch decreased from 8.52 to 6.52 (*P* = 0.0005). The “patient refused” group (*n* = 9) and “per physician” group (*n* = 3) exhibited numeric decreases in pruritus severity, 7.44 to 4.89 and 9.67 to 4.67, respectively.

### Adverse events

A total of 27,495 pre-/post-treatment evaluation notes and 62,386 nursing notes were reviewed. Of this total, 691 pre-/post-treatment evaluation notes and 5979 nursing notes mentioned one of the predefined symptoms and were reviewed manually to determine whether the symptom was present or absent.

Adverse events were rare before and after the first DFK dose (Table 2) for all patients. The percentage of treatments with nausea, diarrhea, vomiting, headache, and trouble walking was similar (*P* ≥ 0.12) before and after the first DFK dose. There were low overall rates of dizziness (before DFK, 0.09%; after DFK, 0.20%) and hyperkalemia (before DFK, 2.0%; after DFK, 2.6%), and these differences were statistically significant. Adverse event information for the CRG and IRG can be found in Supplementary Tables S1 and S2.

**Table 2:** Occurrence of adverse events among all patients before and after DFK administration

All Patients (*N* = 715)			
Adverse Event	Before DFK (Treatments = 27,705) *n* (%)^a^	After DFK (Treatments = 25,141) *n* (%)^b^	*P*-Value
Nausea	234 (0.8)	215 (0.9)	0.8486
Diarrhea	157 (0.6)	162 (0.6)	0.2297
Vomiting	25 (0.09)	34 (0.1)	0.1168
Headache	51 (0.2)	54 (0.2)	0.4122
Dizziness	26 (0.09)	48 (0.2)	0.0027
Trouble walking	0 (0)	0 (0)	—
Hyperkalemia	558 (2.0)	650 (2.6)	0.0001

^a^% of adverse events out all treatments that occurred before DFK

^b^% of adverse events out all treatments that occurred after DFK

DFK, difelikefalin

Among patients who permanently discontinued DFK (*n* = 223), there were few treatments with documented adverse events. However, there were more documented events of diarrhea (before DFK, 0.9%; after DFK, 1.2%; *P* = 0.02), vomiting (before DFK, 0.1%; after DFK, 0.2%; *P* = 0.04), and hyperkalemia (before DFK, 2.2%; after DFK, 3.1%; *P* = 0.01).

Hospitalizations and missed hemodialysis sessions due to any reason were assessed (Supplementary Tables S3 and S4). Both groups experienced an increase in the rate of non-specific hospitalizations (per patient-month) and percentage of missed hemodialysis sessions. However, the non-specific hospitalization rates (per patient-month) for patients in the CRG were lower than rates for patients in the IRG, before DFK (CRG, 0.10; IRG, 0.16) and after DFK (CRG, 0.11; IRG, 0.29). Within the CRG and IRG group, changes in hospitalization rates were not statistically significant before and after DFK (CRG *p* = 0.6259, IRG *p* = 0.1714). A similar pattern was observed for missed hemodialysis sessions, in which the highest percentage of missed sessions was in the IRG, before DFK (CRG, 1.4%; IRG, 5.8%) and after DFK (CRG, 1.8%; IRG 7.8%). There were small but statistically significant differences in missed treatments for the CRG group (*p* = 0.0366), and the IRG group (*p* > 0.0001) before and after DFK.

### Follow-up for change in pruritus severity

**Fig. 2:**
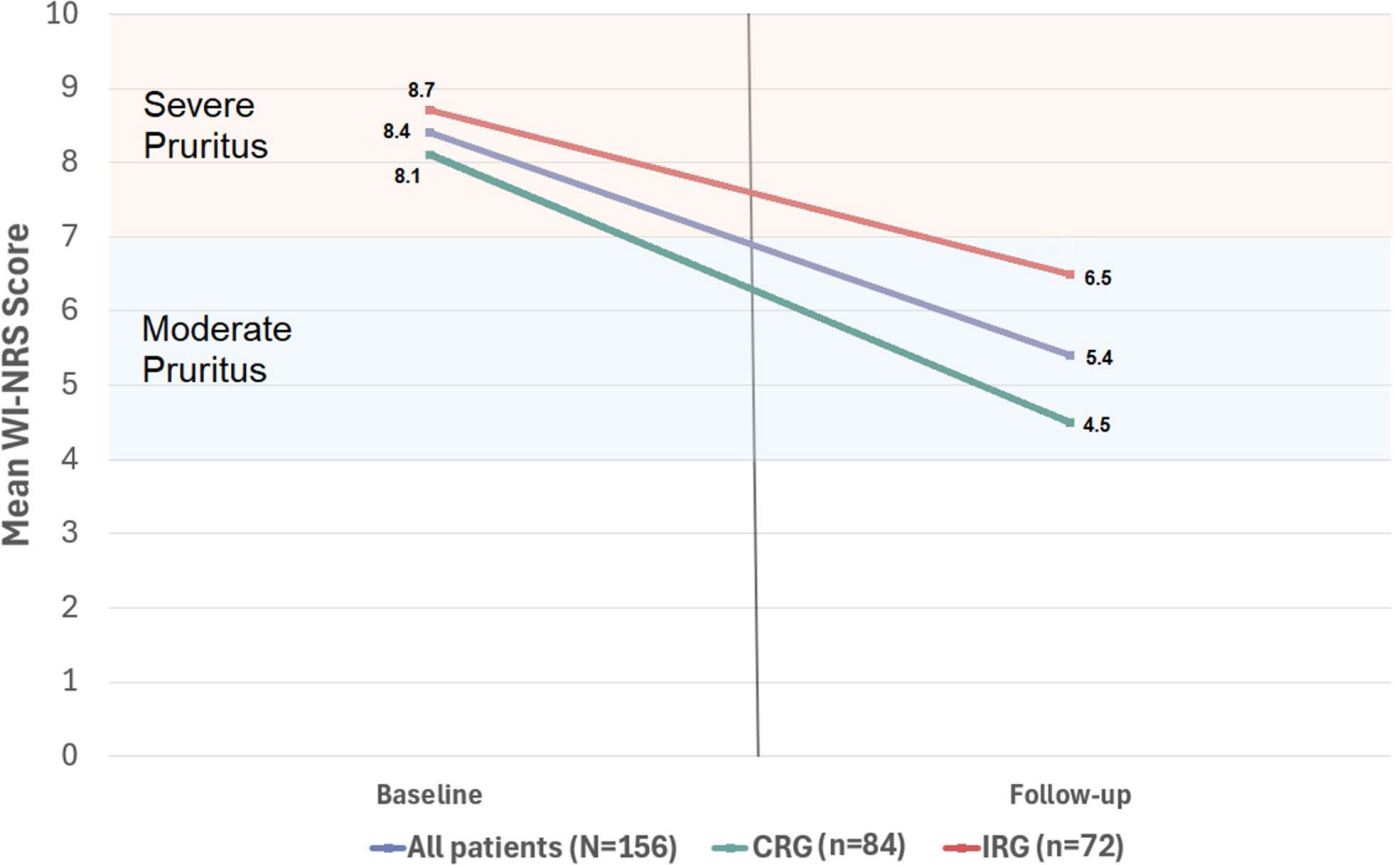
Comparison of baseline and 12-week follow-up WI-NRS scores overall and by subgroup CRG, complete regimen group; IRG, incomplete regimen group; WI-NRS, Worst Itching Intensity Numerical Rating Scale

**Fig. 3:**
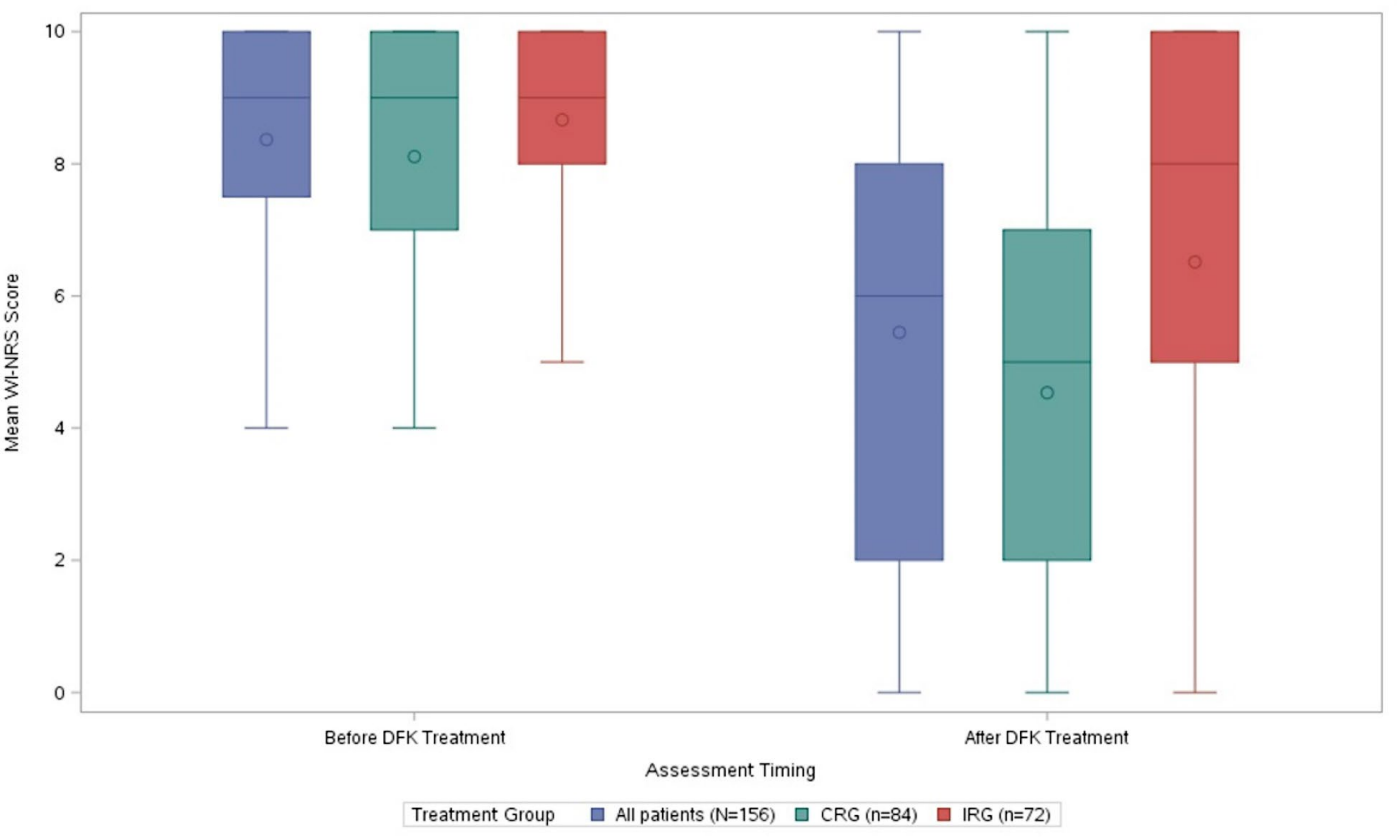
Box plot of baseline and 12-week follow-up WI-NRS scores overall and by subgroup Line within box denotes median, dot denotes mean, and whiskers denote upper and lower bounds. CRG, complete regimen group; DFK, difelikefalin; IRG, incomplete regimen group; WI-NRS, Worst Itching Intensity Numerical Rating Scale

Changes in pruritus ratings between baseline and follow-up for all patients with week 12 data (*N* = 156) and stratified by CRG (*n* = 84) and IRG (*n* = 72) are presented in Figs. 2 and 3. Overall, mean patient WI-NRS scores improved by 2.9 points (8.36 [severe] to 5.44 [moderate]; *P* < 0.0001). This difference was more pronounced among patients in the CRG, who experienced a decrease in itch intensity of 3.6 (8.10 [severe] to 4.53; *P* < 0.0001) compared with patients in the IRG, who experienced a decrease of 2.2 (8.67 [severe] to 6.51 [moderate]; *P* < 0.0001). This difference between groups was statistically significant (*P* = 0.02).

The percentage of patients experiencing 1-, 3-, and 4-point decreases in itch severity is shown in Table 3. Overall, 46% of patients experienced a 3-point reduction in itch severity. More patients in the CRG had a 3-point decrease than did patients in the IRG (56% vs. 35%; *P* = 0.008).

**Table 3:** Comparison of WI-NRS score decreases among patients in the CRG and the IRG

	All Patients (*N* = 156)	CRG (*N* = 84)	IRG (*N* = 72)	*P*-Value (CRG vs. IRG)
WI-NRS decrease by 1+ (%)	68	80	54	0.0006
WI-NRS decrease by 3+ (%)	46	56	35	0.0080
WI-NRS decrease by 4+ (%)	42	48	35	0.1034

CRG, complete regimen group; IRG, incomplete regimen group; WI-NRS, Worst Itching Intensity Numerical Rating Scale

Subanalyses were conducted, stratified by patient treatment with gabapentinoids and antihistamines (Supplementary Table S5). Patients with and without either treatment had similar baseline WI-NRS scores, ranging from 8.66 in patients treated with gabapentinoids at baseline to 8.26 in patients without gabapentinoid treatment. All CRG groups, regardless of gabapentinoid or antihistamine treatment, were observed to have significantly lower pruritus severity following 12 weeks of therapy with DFK (all *P-*values < 0.05).

## Discussion

The present analysis represents the first real-world study of DFK in a US hemodialysis population. During the first year of availability in the United States, more than 700 hemodialysis patients across a large dialysis organization were prescribed DFK for the management of CKD-aP. Although approved for use in patients with moderate-to-severe pruritus associated with CKD-aP in adults undergoing hemodialysis, patients in the present cohort frequently experienced severe symptoms of pruritus before DFK therapy was initiated. As clinical experience with DFK continues to expand, it is reasonable to expect that a broader range of patients may be considered for therapy.

The baseline demographics of the study cohort were similar to those of the broader US hemodialysis population. For instance, the mean age of patients in this analysis was 64.7 years, compared with 63.3 years per DOPPS data [17, 18]. Our cohort had a mean dialysis vintage of 4.8 years, the same as reported by DOPPS [17, 18]. The percentage of patients self-identified as Black or African American in the current cohort was also similar to recent national data from the United States Renal Data System (USRDS) and DOPPS [19, 20].

Among the 156 (22%) of patients with follow-up data, DFK was associated with a mean 3-point reduction in WI-NRS scores. There were 84 patients who met the strict criteria for the CRG, i.e., receiving DFK for approximately 12 weeks. Among these patients, WI-NRS scores were reduced by 3.6 points. 72 patients classified as being in the IRG received DFK less consistently and experienced an average WI-NRS score reduction of 2.2 points. Among the patients for whom DFK was discontinued, we were unable to identify a reason for discontinuation from the medical records in more than 50%. With the exception of the subgroup of patients for whom side effects were noted as the reason for discontinuation, significant reductions in pruritus severity were noted across other discontinuation subgroups. There are many potential reasons why discontinuations may have occurred among patients despite reported reductions in pruritus. It is possible that some patients discontinued therapy (temporarily or permanently) when their symptoms resolved or were reduced. The post-discontinuation nature of pruritus in these patients is not known. Given the high rate of treatment non-completion in the real-world setting, further education for patients and healthcare professionals may be warranted to increase adherence and enhance the assessment of treatment effectiveness. Improved understanding of the clinical and systemic barriers to treatment completion may contribute to better patient outcomes and more robust real-world effectiveness data.

The reduction in WI-NRS scores in the present analysis is consistent with that observed in randomized controlled trials. Overall, we found that 46% of patients experienced at least a 3-point reduction. Such a decrease equates to a change from one severity category to another (e.g., severe pruritus to moderate pruritus). In a pooled analysis of the pivotal trials, 51% of DFK-treated patients reported a ≥ 3 point level of improvement [15]. Among patients in the CRG, 56% reported a ≥ 3 point reduction in pruritus severity and 48% reported a ≥ 4-point reduction in WI-NRS scores. These findings suggest that the results from randomized controlled trials are generalizable to less selected, real-world populations.

The present analysis also adds to the real-world evidence base supporting the use of DFK in patients with moderate-to-severe CKD-aP. Kraft and colleagues recently published data from 15 patients receiving DFK [21]. In this small cohort, they documented a mean 5-point reduction in WI-NRS scores among patients with follow-up data. Although they didn’t detail the duration of follow-up, approximately 73% of their patients continued to receive DFK with every dialysis treatment through October 2022. Consistent with the results from Kraft et al., we also found that DFK significantly reduced pruritus severity among patients receiving concomitant therapies, including gabapentinoids and antihistamines.

Data have demonstrated that patients with increased levels of pruritus are more likely to miss dialysis sessions than patients with a lower symptom burden [22]. In turn, patients with missed dialysis sessions are at increased risk of hospitalization and death [22, 23]. In the present analysis, overall, patients experienced higher rates of hospitalization and missed hemodialysis sessions after DFK was initially prescribed, compared with the pre-DFK period. In the absence of a control group, it is difficult to discern the causative reasons for hospitalization and missed hemodialysis sessions.

The retrospective database nature of our study limits generalizability of the safety findings, but the present data are generally similar to those observed in controlled trials of DFK. In a pooled analysis of the KALM-1 and KALM-2 trials, 6.8% of patients discontinued DFK as a result of treatment-emergent side effects [16]. In the present analysis, 1.5% of patients permanently discontinued DFK as a result of side effects, and an additional 7.5% of patients “refused” to continue treatment (an additional 16% of the cohort discontinued therapy with no reason recorded). In the phase 3 trials of DFK, the most commonly reported treatment-emergent adverse events were diarrhea, dizziness, nausea, gait disturbances, hyperkalemia, headache, somnolence, and mental status changes [16]. In the present study, based on clinical notes, numerically higher rates of nausea (0.9% vs. 0.8%), vomiting (0.1% vs. 0.09%), dizziness (0.2% vs. 0.09%), and hyperkalemia (2.6% vs. 2.0%) were noted in charts after DFK was initiated (vs. before DFK initiation). The clinical notes were assessed for symptoms at all time points, not just at the first dose administration, and all fields in the database were manually reviewed for data collection. We acknowledge that clinical notes may underreport the true incidence of certain conditions. However, our study reviewed clinical notes before and after the treatment period to capture a comprehensive picture of patient experiences and outcomes.

The present analysis represents the first real-world data from a large dialysis organization examining the safety and effectiveness of DFK. With 715 patients, the DFK-treated population in our study is larger than the DFK arms in both phase 3 clinical trials combined (*N* = 426). In the 156 patients (22%) with follow up data, a clinically meaningful reduction in pruritus severity following initiation of DFK was observed.

This study has important limitations. The retrospective and uncontrolled nature of our study precludes any conclusions regarding causality between DFK therapy and symptom reduction. We also cannot account for any other changes in therapy, lifestyle, comorbid conditions, or dialysis parameters that might have contributed to changes in pruritus symptoms or to the frequency of follow-up assessments of WI-NRS. As this was a retrospective analysis, the frequency of follow-up WI-NRS assessments was not under our control and approximately 22% of follow up WI-NRS scores were available. The direction of the selection bias this may introduce is unknown and observed results can not be assumed to be generalized to patients without scores. In addition, the variability in CKD-aP symptom severity over time complicates the assessment of treatment efficacy and underscores the need for more personalized therapeutic approaches [24].

In conclusion, among hemodialysis patients with moderate-to-severe pruritus, initiation of DFK was associated with significant reductions in CKD-aP as measured by WI-NRS, a validated patient-reported outcome measure of pruritus. The incidence of adverse events was low. Those patients remaining on therapy for 12 weeks experienced greater symptom reductions and lower rates of adverse events than those patients receiving partial treatment during the same follow-up period.

Although further studies are needed to evaluate the effects of longer-term treatment with DFK and to further understand the clinical profile of patients who discontinue therapy, these results complement the results of prior controlled trials and suggest that DFK, when administered according to the recommended regimen, is an effective and safe treatment for the management of CKD-aP in patients receiving hemodialysis. This analysis suggests that treatment as per label ensures more pronounced itch relief than that received with incomplete dosing regimens. A better understanding of the IRG is needed for better patient support.

## Electronic supplementary material

Below is the link to the electronic supplementary material.

Supplementary Material 1

## Data Availability

The data underlying the findings described in this manuscript are proprietary and not publicly available. Further inquiries can be directed to Dr. Michael S. Anger at Michael.Anger@freseniusmedicalcare.com.

## Abbreviations

CKDaP: Chronic kidney disease–associated pruritus
CRG: Complete regimen group
DFK: Difelikefalin
DOPPS: Dialysis Outcomes and Practice Patterns Study
ESKD: End-stage kidney disease
FDA: US Food and Drug Administration
FKC: Fresenius Kidney Care
IRG: Incomplete regimen group
NEIRB: New England Institutional Review Board
SD: Standard deviation
USRDS: United States Renal Data System
WI-NRS: Worst Itching Intensity Numerical Rating Scale
