# Supplementary material for: Real-world use of difelikefalin in hemodialysis patients at a large dialysis organization in the United States: a retrospective database study
Source: BMC Nephrol. 2025 Mar 27;26:156. doi: 10.1186/s12882-025-04074-7 (PMC11948630; doi:10.1186/s12882-025-04074-7)
Supplement: Supplementary file 1 — Supplementary Material 1 [file 12882_2025_4074_MOESM1_ESM.docx]

**Supplementary Material**

Supplementary Table S1. Occurrence of adverse events among patients in the CRG before and after DFK administration

Supplementary Table S2. Occurrence of adverse events among patients in the IRG before and after DFK administration

Supplementary Table S3. Rate of hospitalizations before and after DFK administration

Supplementary Table S4. Occurrence of missed HD sessions before and after DFK administration

Supplementary Table S5. Comparison of WI-NRS scores before and after DFK administration among patients stratified by concomitant use of gabapentinoids or antihistamines

**Supplementary Table S1** Occurrence of adverse events among patients in the CRG before and after DFK administration

| **COMPLETE REGIMEN GROUP (CRG) (*N* = 295)** | | | |
| --- | --- | --- | --- |
| **Adverse Event** | **Before DFK**  **(Treatments = 11,052) *n* (%)** | **After DFK**  **(Treatments = 10,806) *n* (%)** | ***P*-Value** |
| Nausea | 64 (0.6) | 64 (0.6) | 0.8984 |
| Diarrhea | 48 (0.4) | 29 (0.3) | 0.0374 |
| Vomiting | 8 (0.1) | 11 (0.1) | 0.4607 |
| Headache | 26 (0.2) | 18 (0.2) | 0.2574 |
| Dizziness | 10 (0.1) | 25 (0.2) | 0.0092 |
| Trouble walking | 0 (0) | 0 (0) | — |
| Hyperkalemia | 202 (1.8) | 264 (2.4) | 0.0019 |

CRG, complete regimen group; DFK, difelikefalin.

**Supplementary Table S2** Occurrence of adverse events among patients in the IRG before and after DFK administration

| **INCOMPLETE REGIMEN GROUP (IRG) (*N* = 420)** | | | |
| --- | --- | --- | --- |
| **Adverse Event** | **Before DFK**  **(Treatments = 14,671) *n* (%)** | **After DFK**  **(Treatments = 12,408) *n* (%)** | ***P*-Value** |
| Nausea | 170 (1.2) | 151 (1.2) | 0.6592 |
| Diarrhea | 109 (0.7) | 133 (1.1) | 0.0042 |
| Vomiting | 17 (0.1) | 23 (0.2) | 0.1379 |
| Headache | 25 (0.2) | 36 (0.3) | 0.0384 |
| Dizziness | 16 (0.1) | 23 (0.2) | 0.0990 |
| Trouble walking | 0 (0) | 0 (0) | — |
| Hyperkalemia | 356 (2.4) | 386 (3.1) | 0.0117 |

IRG, incomplete regimen group; DFK, difelikefalin.

**Supplementary Table S3** Rate of hospitalizations before and after DFK administration

|  | | **BEFORE DFK** | | | **AFTER DFK** | | |
| --- | --- | --- | --- | --- | --- | --- | --- |
| **Group** | ***N*** | **Total Patient Time (mo)** | **Total Hosps** | **Hosps/ Patient-Month** | **Total Patient**  **Time (mo)** | **Total Hosps** | **Hosps/ Patient-Month** |
| All patients | 715 | 1995.7 | 269 | 0.13 | 1880.2 | 395 | 0.21 |
| CRG | 295 | 838.4 | 83 | 0.10 | 844.9 | 90 | 0.11 |
| IRG | 420 | 1157.3 | 186 | 0.16 | 1035.3 | 305 | 0.29 |

DFK, difelikefalin; hosp, hospitalization; CRG, complete regimen group; IRG, incomplete regimen group.

**Supplementary Table S4** Occurrence of missed HD sessions before and after DFK administration

|  | | **BEFORE DFK** | | | **AFTER DFK** | | |
| --- | --- | --- | --- | --- | --- | --- | --- |
| **Group** | ***N*** | **Total Completed HD Sessions** | **Total Missed HD Sessions** | **% Missed/ Expected HD Sessions** | **Total Completed HD Sessions** | **Total Missed HD Sessions** | **% Missed/ Expected HD Sessions** |
| All patients | 715 | 25,723 | 1055 | 3.940 | 23,214 | 1243 | 5.082 |
| CRG | 295 | 11,052 | 157 | 1.401 | 10,806 | 198 | 1.799 |
| IRG | 420 | 14,671 | 898 | 5.768 | 12,408 | 1045 | 7.768 |

HD, hemodialysis; DFK, difelikefalin; CRG, complete regimen group; IRG, incomplete regimen group.

**Supplementary Table S5** Comparison of WI-NRS scores before and after DFK administration among patients stratified by concomitant use of gabapentinoids or antihistamines

| **Group** | ***N*** | **Baseline  WI-NRS Score** | **Follow-Up  WI-NRS Score (Week 12)** | ***P*-Value** |
| --- | --- | --- | --- | --- |
| **+ GABAPENTINOIDS** | | | | |
| All patients | 41 | 8.66 | 6.10 | <0.0001 |
| CRG | 17 | 8.65 | 5.47 | 0.0016 |
| IRG | 24 | 8.67 | 6.54 | 0.0076 |
| **NO GABAPENTINOIDS** | | | | |
| All patients | 115 | 8.26 | 5.22 | <0.0001 |
| CRG | 67 | 7.97 | 4.30 | <0.0001 |
| IRG | 48 | 8.67 | 6.50 | 0.0003 |
| **+ ANTIHISTAMINES** | | | | |
| All patients | 24 | 8.38 | 6.83 | 0.0190 |
| CRG | 10 | 8.30 | 5.60 | 0.0069 |
| IRG | 14 | 8.43 | 7.71 | 0.4113 |
| **NO ANTIHISTAMINES** | | | | |
| All patients | 132 | 8.36 | 5.20 | <0.0001 |
| CRG | 74 | 8.08 | 4.39 | <0.0001 |
| IRG | 58 | 8.72 | 6.22 | <0.0001 |

WI-NRS, Worst Itching Intensity Numerical Rating Scale; DFK, difelikefalin; CRG, complete regimen group; IRG, incomplete regimen group.
